# Children’s lived experience and perceptions of community members’ influence on their schooling: a study in Uganda

**DOI:** 10.3389/fpsyg.2023.1045737

**Published:** 2023-07-19

**Authors:** Richard Balikoowa, Deborah Ojiambo, Lydia Emuron, Godfrey Siu, Christine Mbabazi-Mpyangu, Julius Fred Kikooma, Joshua Mugambwa, Rachael Daphine Nuwagaba, Martin M. Baluku, David Onen

**Affiliations:** ^1^Department of Educational, Social and Organizational Psychology, School of Psychology, College of Humanities and Social Sciences, Makerere University, Kampala, Uganda; ^2^Directorate of Higher Degrees, Kampala International University, Kampala, Uganda; ^3^School of Public Health, College of Health Sciences, Makerere University, Kampala, Uganda; ^4^Department of Religious Studies, School of Liberal and Performing Arts, College of Humanities and Social Sciences, Makerere University, Kampala, Uganda; ^5^Directorate of Research and Graduate Training, Makerere University, Kampala, Uganda; ^6^Faculty of Management, Makerere University Business School, Kampala, Uganda; ^7^East African School of Higher and Development, Makerere University, Kampala, Uganda

**Keywords:** qualitative, students’ perceptions, primary school, community engagement, Africa

## Abstract

**Introduction:**

Global and national initiatives have successfully increased access to public education in low- and middle-income countries. However, many students in rural regions in these countries have high rates of absenteeism and drop-out, and low levels of academic engagement. Together, these significantly limit children’s academic performance and achievement. One strategy that addresses these barriers seeks to engage members of the wider local community in schools. Most previous research focuses on adults’ perspectives even though the potential benefit of community engagement is greatest when learners perceive it to be positive. Past research has also focused on community members structured engagement in activities within schools. This research provides a wider exploration of children’s lived experience and perceptions concerning community members’ influence on their schooling and learning. The aim was to gain a rich picture of how primary school students in rural Uganda perceive that community members’ behavior helps or hinders their education.

**Methods:**

Qualitative data from students 10 to 13 years of age were collected through individual interviews that used the draw-and-talk technique (*n* = 20) and four focus group discussions.

**Results:**

Seven broad categories of community members’ actions were perceived to be positive (conflict resolution; supporting students’ physical well-being; encouraging learning and positive behavior; reducing truancy; providing food and financial support; collective community work; and political representation). Four categories of community members’ actions were perceived to have a negative influence (creating barriers to attending school; noise and other distractions; insecurity; and theft and vandalism).

**Discussion:**

We conclude that carefully structured community involvement in schooling may improve the emotional and material support that facilitates students’ continued school attendance and their motivation for learning. However, we also identify some limits on the role that local communities may be able to play in overcoming the challenges facing education in low- and middle-income countries.

## 1. Introduction

Over the past 20 years, there has been a large increase in the percentage of children in low- and middle-income countries (LMICs) enrolled in school (Ganimian and Murnane, 2016; Bold et al., 2017; Conn, 2017; Evans and Mendez Acosta, 2021). However, this has not been accompanied by a proportionate increase in children’s academic skills (Itegi et al., 2021; World Bank, UNESCO, UNICEF, 2021). More than 60 percent of primary school children in LMICs fail to achieve minimum proficiency in literacy and numeracy skills (Mahuro and Hungi, 2016; World Bank, 2018). The lowest levels of skills acquisition have been reported in sub-Saharan Africa (World Bank, 2018), especially among children living in rural areas (du Plessis and Mestry, 2019; Mukuna and Aloka, 2020; Pillay, 2021; Lian et al., 2023). In addition, many students drop out of school before completing their primary or secondary schooling cycles (Allen and Chavkin, 2004; UNICEF, 2014).

The low level of academic performance and educational achievement in LMICs has been characterized as a learning crisis, due to its long-term consequences for individuals and nations (Allen and Chavkin, 2004; World Bank, 2018). For individuals, gaining literacy and numeracy skills positively affects employment, earnings and health. For nations, these skills facilitate economic growth, reduce poverty, strengthen institutions, and foster social cohesion (World Bank, 2018). Four proximal factors that contribute to the crisis have been identified: unskilled and unmotivated teachers; unprepared learners; school inputs that are ineffective in promoting teaching and learning; and school management that is ineffective in promoting teaching and learning (World Bank, 2018). Due to the diversity of circumstances that underlie these barriers, they cannot be overcome without attitudinal and large-scale structural changes that cannot be achieved quickly and require concerted effort from multiple stakeholders (Ganimian and Murnane, 2016; Mahuro and Hungi, 2016; Islam, 2017; Tindyebwa Muhangi, 2019; Randolph, 2022; Siy Van et al., 2022). The level of community organization at which stakeholders participate differs across countries (Barr et al., 2012; Baquedano-López et al., 2013).

Most previous research on community involvement in children’s schooling and learning has been conducted in high-income countries and has focused on one of three aspects. First, it has focused on specific stakeholders. Most studies have focused on educational involvement by students’ parents. Even studies that purport to also include involvement by members of the wider community usually present data that almost exclusively relates to parents (Van Roekel, 2008). Parental involvement takes diverse forms including upgrading school facilities; monitoring school leadership; promoting quality learning programs; provision of resources to improve teaching; extra funding for after-school programs; and sponsorship of peer-to-peer learning programs (Henderson and Mapp, 2002; Najjumba et al., 2013; Spier et al., 2016). Second, research has focused on community members’ involvement in interventions that target specific school activities such as music, reading and sports (Camiré et al., 2012; LaVenia and Burgoon, 2019; Prest, 2020) or general academic subjects (Invernizzi and Juel, 1996; Jackson and Hosch, 2002; McLurkin, 2006; Holt and Asagbra, 2021). Lastly, some research on community involvement in schools focuses on a specific type of school, “community schools,” that exist in some high-income countries, such as the USA (e.g., Epstein, 2018; Daniel et al., 2019).

There is relatively little research on the many other ways in which communities support children’s schooling in LMICs (Humble and Dixon, 2017; Iyengar, 2021; Siy Van et al., 2022). Diverse initiatives that involve members of the wider community in supporting students and schools have been implemented in many countries in Africa, South America and Asia (Israel et al., 2001; Hohlfeld et al., 2010; Parkes et al., 2016; Spier et al., 2016; Itegi et al., 2021; Salac and Florida, 2022). For example, a variety of innovative programs in which community members supported student schooling during COVID-19 school closures were implemented in LMICs as well as in high-income countries (Power et al., 2021).

Evaluations of the effectiveness of interventions promoting community involvement in schools in high-income countries have shown inconsistent findings (Sanders, 2001a,b; Sanders and Epstein, 2013). The outcome measures in most evaluations has been students’ test scores or adults’ perceptions (e.g., Kawa, 2022; Randolph, 2022; Mukhopadhyay, 2023). While many factors are likely to contribute to the effectiveness of interventions, it seems likely that community members and activities that students perceive to be acceptable and effective will have the greatest impact (e.g., Fall, 2006; Reeves and Gomm, 2015). However, there is a dearth of research on children’s perceptions of community members’ influence on their schooling.

Uganda presents a useful context in which to study students’ perceptions of community involvement in their schooling. In 1997, the Government of Uganda introduced Universal Primary Education (UPE) and abolished school tuition fees in government-funded primary schools to ensure that a basic education was affordable (Oketch and Rolleston, 2007; Itaaga et al., 2014; ODI, 2021). Subsequently, many more girls and children from poor households enrolled in school. The primary school population doubled in a single year (1996 to 1997), but the national education budget increased by less than one-third (Nishimura et al., 2008; Kasirye, 2009). As a result, one-quarter of the students who completed the standardized primary leaving examination received a failing score (Deininger, 2003).

In the years since, the number of primary schools and qualified primary school teachers has dramatically improved. However, due to population increases, the population of primary school students has also dramatically increased, from approximately 3 million students in 1998, to over 8 million in 2015 (Datzberger, 2018; ODI, 2021). As a result, there continues to be a national shortfall in infrastructure, teaching and learning resources, and qualified teachers (National Planning Authority Uganda, 2015). In rural areas, many children remain out of school (UNICEF, 2014), and those children who attend school continue to have low levels of achievement and high levels of drop-out (e.g., Epstein and Sheldon, 2002; Sabates et al., 2010). Students in Uganda have weaker academic performance than students in many other African countries (Busingye and Najjuma, 2015; Okello et al., 2020; Sekiwu, 2020), with students from rural areas having particularly poor performance (Acham et al., 2012; Nakabugo, 2015; Mukuna and Aloka, 2020; Okello et al., 2020; Lubaale et al., 2021; ODI, 2021). Additionally, Uganda is reported to have one of the highest primary school dropout rates worldwide, with estimates that as few as 32.1 per cent of Ugandan children complete primary education (Nakabugo, 2015; Omoeva and Gale, 2016; Lubaale et al., 2021).

Ugandan government policies explicitly encourage community participation in schools: “communities are expected to be the true owners of schools and therefore are expected to take keen interest then in terms of monitoring school activities and participating in their development” (National Planning Authority Uganda, 2015, p. 13). Such involvement is perceived to be directly relevant to increasing the effectiveness of schooling: “low community participation is one of the major impediment [*sic*] to the realization of quality primary education in the country” (National Planning Authority Uganda, 2015, p. 13). However, government policies provide little guidance about the types of community contribution that are welcome beyond membership of schools’ Parent Teachers Associations and Board of Governors. As a result, community participation in Ugandan schools is often unstructured, inadequate and heavily reliant on government and political interventions (Chavkin, 2000; Aref, 2010; Rukundo, 2018; Tumusiime, 2018; Nelson et al., 2022).

This study addressed the gap in our understanding of the perceptions that students in LMICs hold concerning community members’ influence on their education by seeking insights into their lived experience of community members’ positive and negative influences on their schooling and learning. It explored two research questions:

1. What range of community members do primary school students in rural Uganda perceive as influencing their schooling and learning?
2. What activities by these community members positively or negatively influenced their schooling and learning?

## 2. Methods and materials

### 2.1. Study design and context

We adopted a multi-site cross-sectional qualitative research design. The study was conducted in two schools in each of two parishes (Walibo and Itakaibolu) in Luuka, a rural district in Waibuga sub-county in Eastern Uganda. This location was chosen because of its persistent low levels of school achievement, and students’ irregular school attendance and participation (Kirunda and Okeya, 2013; Walukamba, 2013; Emodek, 2017). For example, for the past two decades, both primary and secondary schools in the region have consistently achieved poorer results in national examinations than schools in other regions of Uganda (Itaaga et al., 2014). The area is characterized by widespread socio-economic disadvantage (Abbo et al., 2008; Wafula, 2020).

### 2.2. Participants

Primary school students (interviews: *n* = 20, 50% female; focus groups: *n* = 4; 50% female) were purposively selected from four government-funded schools. Grade 4 and Grade 5 students (10 to 13 years of age), who had been residents of the local community for at least two years were recruited. This age group was selected to ensure that participants had the ability to describe their lived experiences in detail, and the residency requirement ensured that participants had the opportunity to experience diverse influences from community members.

### 2.3. Ethical considerations

All the participants were recruited after ethical approval and in strict conformity to the guidelines provided by the Gulu University Research Ethics Committee (GUREC) and the Uganda National Council for Science and Technology (UNCST). Furthermore, all members of the research team had undergone training in child-related research ethics, and they continued to obtain guidance throughout the study period. If children expressed emotional disturbance as they disclosed their experiences, they would be referred to specific teachers who had been given guidance by the child-counseling expert on the research team. School authorities facilitated the distribution of consent forms to parents. Students who agreed to participate in the study received consent forms in the area’s local language (Lusoga) while they were at school and were asked to take them home to their parents/guardians. Students whose parents/guardians provided informed consent were asked whether they assented before participating in the research. All participants who assented completed the study, although they were assured that they could withdraw at any time.

### 2.4. Data collection

In-depth individual interviews were complemented by four Focus Group Discussions, two in each school. A child-friendly draw-and-talk method of collecting data was used. Participants were asked to draw their portraits and encouraged to tell stories related to their schooling environment and experiences. They were also asked to draw figures of the community members whose activities had influenced their schooling and learning, to explain how their activities helped or hindered them. Thus, drawings were used as both data, and as tools that helped children communicate complex information about their experiences and perceptions (Balikoowa, 2023) and made the research process enjoyable for the child participants (Angell et al., 2015).

### 2.5. Analytic framework and process

We made a detailed exploration of individual children’s personal and lived experiences; and how they made sense of the relationship between the activities of members of their community and their learning and schooling (Reid et al., 2005).

All interviews were audio recorded, translated, and transcribed by the data collection teams. Transcripts were cross-checked to ensure completeness and accuracy. After reading through the transcripts and viewing the drawings, we documented general thoughts about the data. We noted statements representing the emerging meaning units. After listing non-overlapping statements, coders proceeded to develop a codebook. Codes were developed using both inductive and deductive processes. That is, some codes emerged from the transcripts while others were informed by past research. After developing the codebook, we synthesized the data by relating and clustering the meaning units or codes into preliminary themes and developed descriptions representing the interpretation of themes. To eliminate redundancy, we followed Miles et al.'s (2014) suggestions by merging preliminar*y* themes to formulate categories of themes. As Creswell (2014) recommended, the validity of coding and theme identification was checked through data triangulation by sharing and comparing emerging codes and themes. The process of coding and the identification of themes was aided by Nvivo11 Professional software.

### 2.6. Dissemination procedures

The results of the research were disseminated to participants and stakeholders through workshops held at both local/district and national levels. Local workshops were attended by student leaders, school administrators and parent representatives. National workshops were attended by officials from the Ministry of Education as well as local and national media.

## 3. Results

### 3.1. Range of community members students perceived to influence their schooling and learning

The children understood their community to include all persons within and around their school environment. That is, their answers included a far broader range of individuals and roles than have been discussed in past research on community engagement in schooling. The community members they perceived to influence their schooling and learning included members of their school community such as student leaders (e.g., head girls and boys), class teachers and head teachers, in addition to diverse members of the wider community, including local spiritual leaders (e.g., pastors, reverends, and imams) and regional spiritual leaders (e.g., bishops), police officers, leaders of local councils (from Local Council 1 to Local Council Five) and politicians who represented the area in the national government, health workers (e.g., nurses, doctors), and shopkeepers.

### 3.2. Students’ perceptions of community members’ positive influences on their schooling and learning

#### 3.2.1. Conflict resolution

The students identified several circumstances in which a community member helped to resolve a conflict involving their school. For example, they reported that the chairman of Local Council One (LC1) had an indirect influence by helping to resolve disagreements within their community that could ultimately hinder their school attendance or learning:

“…the LC1 solves the conflicts among the village members, and the village will be at peace…” (Focus Group Discussion 1).

This councilor also had a more direct role on their schooling by helping to resolve conflicts between the school and the owners of neighboring properties:

“…when we go to our school field to play, this neighbour chases us away, saying we play in his garden. The school neighbour claims that this land, where our school pitch is located, is his. Therefore, he does not want the school children to play from this land…but this LC1 chairman comes and calls a meeting…and people in the meeting tell the neighbour to leave children using the playground…” (Focus Group Discussion 1)

The students also recognized that the head teacher at their school played an important role in their education by settling disputes between students:

“…the head teacher also helps to settle conflicts in our classes when children are fighting”… (Focus Group Discussion 1)

Other comments from the children acknowledged the role that their community leaders played in protecting their school property, including land and buildings.

#### 3.2.2. Supporting students’ physical well-being

Unlike international research on community members’ support for children’s schooling, the students in this study recognized the important role that health care played in enabling them to attend school and continue their learning when they experienced minor health issues. They specifically mentioned the treatment a nurse and the head girl provided for menstrual-related pain, fever, and minor injuries:

“[…] this nurse (in the drawings) lives in the town centre near our school. She treats school children when sick, even when they get wounds after knocking their toes while walking to school”…

“…yes, even when some girls feel abdominal pains due to their (menstrual) periods, the head girl and the nurse help them.” (Two students in Focus Group Discussion 1)

The students added that medical treatment that speeded their recovering when they became seriously unwell also supported their education:

“…so when you get well, then you can study….” (13-year-old male student in Focus Group Discussion 2)

Student leaders also supported the students’ education through prevention and health promotion initiatives, for example by encouraging them to be clean and protect themselves against infections and diseases at school:

“She [head girl] also encourages us to be clean at school ….” (Focus Group Discussion 1)

In addition, students reported that some members of the wider community spontaneously provided support for their physical wellbeing while they engaged in school activities:

“…the woman in this picture here sells pancakes near our school, but she also helps us with some water when we go for athletics and other field activities…it helps us to cure from the thirst ….” (10-year-old female student in Focus Group Discussion 1)

#### 3.2.3. Encouragement for learning and positive behavior

Students indicated that members of the wider community contributed to their schooling in ways that are consistent with the policies of the Ugandan government and previous research. For example, they attended school meetings during which they formulated policies and deliberated on issues that affect children’s schooling. In addition, Focus Groups 1 and 4 discussed the ways in which diverse community members encouraged school attendance. These community members included police officers, and local (e.g., reverends and imams) and regional (e.g., bishop) religious leaders.

However, the students also showed a broader conception of both their community, their learning, and the types of support they received. Although international literature on community members’ involvement in children’s education excludes the role of teachers, students in the current study perceived school staff to be integral members of their community. They therefore found it natural to include teachers among the community members who contributed to their education. Teachers supported students’ academic learning and encouraged positive behavior as part of their wider education:

“…teachers teach children…they are members of our community that help with our learning.” (Focus Group Discussion 3)

“… the teacher tells us to know how to behave… he helps us to be good leaders at school …” (Focus Group Discussion 2)

“… the teacher guides children to participate in games like football (soccer), netball, athletics, music and others…he also teaches us to behave well and to have discipline….” (12-year-old male student)

Thus, students perceived their learning to be multidimensional, with academic subjects comprising only one of these dimensions. They understood that they were also being enculturated into community norms of behavior. Diverse community members were identified as counseling the students to respect each other, other people at school, and other community members by behaving well, having discipline, and avoiding insulting or abusing others. Community elders, in particular, played a role in discouraging the students and other young people in the community from fighting. Leaders from different religious traditions also contributed to this dimension of learning:

“…the Imam teaches us not to steal other people's properties …. and the Pastor teaches us not to abuse others …He also teaches us to respect others …” (Focus Group Discussion 4)

Although much of this instruction occurred during everyday interactions or community events, it sometimes occurred when members of the wider community visited the school to address the students:

“…. in this picture is a woman who works as a member of the women (council) leaders. Here she has been invited by the school and is encouraging children, especially the girls, to dress well, to kneel and greet people they meet…” (12-year-old male student in an individual interview)

Community members also supported children’s schooling in ways that are rarely a focus of research in high-income countries. First, they explicitly acknowledged spiritual support. In particular, the participants mentioned that the Bishop prays for the children when they are going to sit for their exams. Second, community members discouraged students from being drawn into child labor. Teachers, community leaders and religious leaders encouraged primary school students to continue studying rather than becoming employed in cutting sugarcane, the area’s greatest economic activity:

“…teachers advise children to study…not to go to cut sugarcane….” (Focus Group Discussion 3)

“…and Reverends tell children not to go and cut sugarcane and also not to steal….” (Focus Group Discussion 4)

#### 3.2.4. Reducing truancy

Students reported that local police officers actively attempted to reduce truancy:

“… here, a police officer gets children from the market, those that miss classes and go to the market to watch movies in local movie halls …the police brings them back to school ….” (Focus Group Discussion 1)

The police also reduced truancy when they enforced laws to prevent child labor:

“…If you have gone to cut sugarcane, police can arrest you and put you to order….” (12-year-old male student)

#### 3.2.5. Providing food and financial support

Community members supported students learning by filling gaps in material resources resulting from widespread poverty. For instance, students revealed that head teachers pay fees for some children at school. Such financial support is also sometimes provided by members of students’ extended family or members of the wider community, including mechanics. In addition, some students in public primary schools that were originally founded by churches continue to receive highly valued food donations from church members:

“…when we do not have money, this man goes and sells water and gives us money…; they pay our school fees, buy us pens …. there are times when these people here, the church leaders give us maize for porridge. We sometimes eat posho they provide us at school…they also give us some water for drinking when we are thirsty….” (12-year-old student)

#### 3.2.6. Collective work by communities

Because most past research has been conducted in high-income countries, it has not addressed community members’ role in ensuring children have access to the basic infrastructure necessary for them to reach school. Students’ school attendance was aided by collective work that community members completed to maintain or improve basic infrastructure. Many of the children attending schools in these rural districts need to trek to school along paths between villages, which are constantly encroached by vegetation. When speaking of community work groups, one student said

“…they slash the bushy areas on the roads, and we can have good roads when we go to school….”

#### 3.2.7. Political representation

Politicians’ role in the development of infrastructure that supported school attendance was also acknowledged. The local Member of Parliament (MP) had been effective in gaining improved roads through presenting the development needs of the students’ communities to the national parliament:

“…the MP helps us by taking (presenting) our needs to Parliament and get them… like our roads when they are in bad shape, and the President makes (constructs) them for us…”

Local politicians had also been instrumental in providing infrastructure necessary for school attendance:

“…also, our local council representatives, like district councillors, come here to our school meetings, and after delivering our message, we pass it on through singing to the district authorities, and the district responds. For example, Kyankuzi [a swamp] floods during Primary Leaving Examinations and some children cannot cross to school. However, our Parish councilor (name withheld) reported to the district council, and the bridge was constructed to enable us to cross to school during rainy season…”

### 3.3. Students’ perceptions of community members’ negative influences on their schooling and learning

Children were also permitted to share what they perceived to be unhelpful community influences on their schooling and learning.

#### 3.3.1. Creating barriers to attending school

A few students reported that the chores imposed by some guardians limited children’s opportunity to attend school:

“Some step-mothers make children babysit babies, and they miss class”

“… they also give them much work at home to do, and children reach late at school…” (Focus Group Discussion 1)

In some cases, guardians also stopped children from attending school.

#### 3.3.2. Noise and other distractions

Rural schools do not have infrastructure that is insulated from noise. The students reported that noise created by activities by some community members distracted them from learning:

“…the mechanics knock, and we cannot hear when they are teaching.” (Focus Group Discussion 1)

“…these drivers of cars, they drive around school and make noise for us as we are studying, and this makes children lose concentration…” (Focus Group Discussion 2)

Other activities by community members promoted truancy:

“…they bring music near our school… much noise comes from the very loud speakers… sometimes, some school children follow the music and so miss school activities…” (Focus Group Discussion 2)

#### 3.3.3. Insecurity

Students reported that they were vulnerable to sexual, physical and spiritual assault from some community members, especially on their way to and from school. Fear of sexual assault was reported by female students:

“…boys and men rape girls when they go back home… they gear at them, sometimes when the girls are walking through the town centres… this makes us fear coming to school for some school activities. We cannot even come to school for extra academic activities like evening reading …they also take drugs …” (12-year-old female student in Focus Group Discussion 1)

Both boys and girls feared kidnapping. Across a period of more than 20 years, the rebel group the Lord’s Resistance Army, abducted over 25,000 school-age children and youth, mainly in Northern Uganda (Annan et al., 2006; Pham et al., 2008). Although kidnapping is currently rare, it remains a realistic fear among school children:

“The kidnappers rape girls on the way back home; they cut off children’s heads, and this scares us when we are going back home. They [kidnappers] scare children when we are going back …” (Focus Group Discussion 4)

Based on some highly publicized accusations, the students in two focus groups perceived that travelling to and from school also placed them at risk of witchcraft and kidnapping:

“…witch doctors cut off children's heads, and this scares us…the witch doctors murder people and take their heads for riches” (Focus Group Discussion 3)

Many of the participants whose route to school used paths that wind through sugarcane plantations reported feeling unsafe due to the unpredictable behavior of plantation workers who used drugs:

“… I do not feel safe, especially while walking to school via this sugarcane plantation…we smell the drugs being smoked by some men and young boys who dropped out of school,” “…they sometimes force you to take drugs.” (Focus Group Discussion 4)

#### 3.3.4. Theft and vandalism

Students reported that a variety of forms of theft directly or indirectly affects their schooling. In some cases, the students were victims of theft:

“… Those thieves, if they get you when they have sent you to the shop or if you are going to buy things to use at school, they take away the money that you have, sometimes by force …they grab the money from you …” “They wait for us when we return home… Take our money and bags…” (Focus Group Discussion 3)

In other cases, parents were the victims:

“…they steal people's property… even from parents … meant for our school fees…” “*…*thieves steal money from the parents” (Focus Group Discussion 3)

The students also stated that some community members vandalize school property, including damaging the windows and doors of classrooms, and the flowers, trees and fences in their school compounds. They attributed some of this vandalism to drunkenness:

“[they] become very destructive when they get drunk ….” (Focus Group Discussion 4)

## 4. Discussion

With the support of child-friendly data collection procedures, primary school students in rural Uganda were able to provide a rich and nuanced description of the variety of ways in which members of their community influenced their schooling and learning. Their drawings, narratives and comments during focus group discussions reported that a far wider range of community members influenced their schooling and learning than has been captured in previous research, which has typically focused more narrowly on structured forms of community engagement that occur within schools (e.g., Rose, 2003; Wilson and Kolander, 2003; Fabunmi, 2005; Marope and Sack, 2007; Nakpodia, 2011; Van Wyk and Marumoloa, 2012; Islam, 2017). The students’ broader conception of their learning, encompassing activities both inside and outside school, also contributed to the larger number of community members they identified. The students’ reported a correspondingly wide range of influences on their schooling and learning. They focused on seven broad categories of positive direct and indirect influences (conflict resolution; support for students’ physical wellbeing; encouragement for learning and positive behavior; reducing truancy, providing food and financial support; collective work by community members; and political representation). They also identified four broad ways in which community members exerted a negative influence on their schooling and learning (creating barriers to attending school; noise and other distractions; insecurity; and theft and vandalism).

Despite the broader scope of their responses, the students also reported on one aspect of community engagement that has been a focus of previous research on school engagement: resource mobilization (Tindyebwa Muhangi, 2019; Mgaya, 2021; Nguyen and Nguyen, 2021). The students particularly valued the contribution of food to the school meals program. Many previous studies have highlighted the importance of adequate nutrition for learning among students in LMICs (Acham et al., 2012; Najjumba et al., 2013; Balikoowa, 2014; Wang et al., 2021).

Roekel wrote, “…It takes a village to raise a child is a popular proverb with a clear message: the whole community has an essential role in the growth and development of its young people” [2008, p. 1]. The potential benefit of community engagement appears greatest when children perceive this to be positive (Hart, 2013; Reeves and Gomm, 2015). The current findings demonstrate the significance students attributed to community members’ influence on their schooling and learning, and add to evidence that community involvement can facilitate students’ ongoing engagement in schooling by contributing to their emotional and material support (Ashouri and Rasekhi, 2016; Smith et al., 2022). Moreover, although the students in the current sample identified a wide range of community members who exerted very diverse positive influences on their education, they did not mention some types of community engagement that have been the focus of research in high-income countries. These include local musicians’ involvement in school music programs, storytelling by elders, and older athletes serving as coaches for school sports. This suggests that there is scope for the expansion of community engagement in the communities in which this study was conducted. It sems likely that this is also true in other contexts in LMICs. Thus. interventions that increase the level, breadth and effectiveness of community support for students’ schooling and learning appear to hold the potential to improve the sustainability, equity and inclusiveness of access to basic education in rural communities in Uganda and other low-resource settings.

However, previous research sounds a note of caution by showing that such initiatives need to be carefully structured if they are to achieve the desired outcomes. Some community engagement programs in high-income countries have been ineffective and/or inefficient in improving students school learning (e.g., Nichols et al., 2020). Community engagement programs can also increase students’ vulnerability to abuse (e.g., Erooga et al., 2020), or perpetuate existing inequities, especially if community engagement is greatest or delivers more resources to students and schools that are already advantaged (Namatende-Sakwa et al., 2023) or to specific subgroups within a school, for example, based on gender, religion or ethnicity (e.g., Brent, 2000; Green, 2017).

Fewer hazards appear to be associated with initiatives to reduce the negative influences of community members identified by students. Previous research has also documented the threat that school students in LMICs face from gender-based (e.g., Perrin et al., 2019) and other forms of violence in and around schools (e.g., Zulu et al., 2004; Thulin et al., 2019). Similarly, vandalism and theft from schools by members of the local community, and drug and alcohol use in the vicinity of school children have also been documented in other LMICs. A range of focused educational (e.g., de Lang and Geldenhuys, 2012) and policing interventions have been trialed (e.g., Van Jaarsveld and Minnaar, 2012). The latter are more resource intensive than most rural villages in LMICs can sustain. However, both approaches may have a place in the short-term because the factors that underlie many of these behaviors are unlikely to be overcome without attitudinal and large-scale structural changes that cannot be achieved quickly and are likely to be achieved too late to benefit the current generation of students.

### 4.1. Strengths and limitations

The main strength of the current study is that its research method allowed students to create their own narratives to describe their lived experience and perceptions, and thereby gave them agency to be the originators, rather than only the subjects, of knowledge. The research method was also successful in producing rich qualitative data. However, many of the limitations of the current research can also be attributed to its research method. It could not provide any data about the effectiveness of community members’ positive activities or the extent to which their negative activities impaired students’ learning or school attendance and participation. No data about school attendance, grades, or students’ behavior were collected. It is therefore not possible to compare the findings of this study with those from earlier quantitative research that examined the relationship between community engagement in students’ schooling and diverse student outcomes. In addition, as with other qualitative research, the findings lack generalizability. For example, the students in the current sample appreciated that head teachers and members of the wider community paid fees to allow some children from poor families to continue to attend school. Given the high rates of poverty in many rural areas of Uganda, it seems likely that students in other areas of the country also have precarious school enrolment. It is unclear whether their head teachers and members of their communities have the motivation or capacity to emulate the altruism observed in the current study. Quantitative data from a representative sample of rural children would be required to determine this. The current study also focused exclusively on children who continued to attend school. It is therefore unclear to what extent negative influences by community members contributed to school drop-out by other children, or what types of positive community influences might have prevented this. Future research might also explore how community members influence children’s learning and schooling in urban centers, which may have distinct dynamics due to the more transient nature of the population and their greater exposure to modernity and individualism (Greenfield, 2009).

## 5. Conclusion

Most rural schools in LMICs have low access to teaching and learning resources. However, the communities in which they are based have strengths that have the potential to improve school attendance and learning outcomes if well-structured and effective strategies to access these strengths can be developed and implemented (Jeynes, 2007; Christenson and Reschly, 2010; LaRocque et al., 2011; Snilstveit et al., 2016). However, it is important to acknowledge that community engagement can play only a limited role in overcoming the challenges faced by school systems in LMICS. First, the communities in which the need for student and school support is greatest are also the communities with the most limited human (e.g., low literacy rates), material and financial resources. Second, communities’ capacity for engagement is instable. The factors that increase the number of students requiring support and the magnitude of the support they require (e.g., epidemics, natural disasters, conflict) are also the factors that deplete the resources their communities have available to meet their needs. Third, community resources are finite. The marshaling of these resources for the support students and schools needs to very carefully structured and efficient to prevent resources being diverted from other vulnerable groups (e.g., widows, young children with a disability, the elderly). Despite these limitations, increasing community engagement in schooling and learning remains an important and feasible strategy for improving children’s educational outcomes in contexts in which national and state governments have limited funds available to address urgent needs across many sectors.

## Data availability statement

The raw data supporting the conclusions of this article will be made available by the authors, without undue reservation.

## Ethics statement

The studies involving human participants were reviewed and approved by Gulu Research Ethics Committee (GUREC). Written informed consent to participate in this study was provided by the participants’ legal guardian/next of kin.

## Author contributions

RB was responsible for the study design, led the team for data collection, participated in data analysis, and drafted the manuscript. All authors contributed to the article’s development and revision and approved the revised version for submission.

## Funding

This publication is part of a baseline child-focused study funded by ChildFund International through AfriChild Centre, Makerere University, under the project titled Child-focused Research Grants under the Interuniversity Research Training Program (grant number 99-0218D). The contents of this article are the sole responsibility of its authors and cannot be taken to reflect the views of ChildFund International nor AfriChild Centre, Makerere University.

## Conflict of interest

The authors declare that the research was conducted in the absence of any commercial or financial relationships that could be construed as a potential conflict of interest.

## Publisher’s note

All claims expressed in this article are solely those of the authors and do not necessarily represent those of their affiliated organizations, or those of the publisher, the editors and the reviewers. Any product that may be evaluated in this article, or claim that may be made by its manufacturer, is not guaranteed or endorsed by the publisher.
